# Seasonal Differences in Extinction and Colonization Drive Occupancy Dynamics of an Imperilled Amphibian

**DOI:** 10.1371/journal.pone.0127059

**Published:** 2015-05-18

**Authors:** Lea A. Randall, Des H. V. Smith, Breana L. Jones, David R. C. Prescott, Axel Moehrenschlager

**Affiliations:** 1 Centre for Conservation and Research, Calgary Zoological Society, Calgary, Alberta, Canada; 2 Wildland Consultants Ltd., Barrington, Christchurch, New Zealand; 3 Environment and Sustainable Resource Development, Fish and Wildlife Division, Red Deer, Alberta, Canada; Smithsonian's National Zoological Park, UNITED STATES

## Abstract

A detailed understanding of the population dynamics of many amphibian species is lacking despite concerns about declining amphibian biodiversity and abundance. This paper explores temporal patterns of occupancy and underlying extinction and colonization dynamics in a regionally imperiled amphibian species, the Northern leopard frog (*Lithobates pipiens*) in Alberta. Our study contributes to elucidating regional occupancy dynamics at northern latitudes, where climate extremes likely have a profound effect on seasonal occupancy. The primary advantage of our study is its wide geographic scale (60,000 km^2^) and the use of repeat visual surveys each spring and summer from 2009–2013. We find that occupancy varied more dramatically between seasons than years, with low spring and higher summer occupancy. Between spring and summer, colonization was high and extinction low; inversely, colonization was low and extinction high over the winter. The dynamics of extinction and colonization are complex, making conservation management challenging. Our results reveal that Northern leopard frog occupancy was constant over the last five years and thus there is no evidence of decline or recovery within our study area. Changes to equilibrium occupancy are most sensitive to increasing colonization in the spring or declining extinction in the summer. Therefore, conservation and management efforts should target actions that are likely to increase spring colonization; this could be achieved through translocations or improving the quality or access to breeding habitat. Because summer occupancy is already high, it may be difficult to improve further. Nevertheless, summer extinction could be reduced by predator control, increasing water quality or hydroperiod of wetlands, or increasing the quality or quantity of summer habitat.

## Introduction

Over recent decades, amphibian populations have experienced declines on a global scale [1]. It is estimated that more than 40% of amphibian species are in decline and population trends are unknown for almost another 30% of species [2]. However, a complete understanding of the metapopulation is lacking for many anuran species. Knowledge regarding seasonal changes in occupancy would help target research to appropriate times of year and identify factors preventing recovery. Understanding the extinction and colonization dynamics can help identify management actions and conservation efforts that are most likely to increase occupancy [3]. An understanding of regional population dynamics is essential to accurately assess annual population trends, evaluate the conservation status of at-risk amphibians [4], and gauge the effectiveness of conservation measures.

Although there are an increasing number of multi-year occupancy studies, broad-scale population monitoring of amphibians is infrequently undertaken due to the prohibitive cost and effort of such programs [5]. For this reason, many studies of amphibian population dynamics have focused on individual and site-specific state variables such as reproductive rate and survival, or abundance. Although changes in these state variables within individual populations likely reveal vital information regarding population trends at single sites, understanding changes in occupancy across a broad spatial scale may be more useful for the conservation of at-risk species [5,6].

Studies of species distribution or abundance are usually conducted at a single time of year, yet habitat requirements may differ by life stage or season. This may introduce uncertainties in the management of key habitat or critical life-stages. For example, occupancy studies may reveal interesting snapshots of species distribution within a single year (e.g. [7,8]) or reveal longer-term dynamics at a single time of year (e.g. [9,10]). However, restricting occupancy studies to single seasons may limit insights into important contributions of both seasonal and annual occupancy (e.g. [11]) or the underlying extinction and colonization dynamics to long-term dynamics (e.g. [12]).

Amphibian species at northern latitudes, including the Northern leopard frog (*Lithobates pipiens)*, have likely evolved strategies or possess adaptations to help cope with extreme seasonal shifts in climate that are likely to influence occupancy dynamics. Northern leopard frogs require three distinct seasonal habitats (spring breeding, summer foraging, and overwintering habitats) [13]. In southern Alberta, Canada, Northern leopard frogs gather at breeding ponds between mid-April and late June and eggs hatch within a few days to up to a month [14,15]. Tadpoles are confined to breeding ponds until metamorphosis occurs at the end of July to mid-August [14]. Long distance dispersal and colonization of new wetlands is primarily by juvenile frogs [16]. Young of year and adult frogs tend to remain near water to escape predators and forage in adjacent fields until September–October when they may migrate along waterways to overwintering sites, located in nearby streams or rivers and other well-oxygenated bodies of water [14]. In some cases, a single water body may meet all of the seasonal habitat requirements. For these reasons seasonal differences in occupancy patterns may be driven by changing habitat requirements and occupancy should be interpreted as “seasonal occupancy” for the purpose of this study.

Although much is known about the development, anatomy and physiology or Northern leopard frogs (e.g. [17,18]), little is known about their occupancy dynamics [13]. Once widely distributed and abundant, Northern leopard frogs vanished from large portions of western North America in the 1970s and 1980s, including much of their historic range in Alberta, Canada, perhaps due to factors such as disease, drought, invasive species, habitat loss and fragmentation [19]. As a result, the western boreal/prairie populations of Northern leopard frog are designated as *Special Concern* by the Committee on the Status of Endangered Wildlife in Canada [19] and *Threatened* under the Alberta Wildlife Act [13]. Recovery efforts in Alberta have focused on reintroducing Northern leopard frogs to parts of their former range and protecting habitat [13], but the success of these initiatives is still uncertain. It remains unclear whether Alberta populations continue to decline, have stabilized at current levels or have begun to recover.

This paper is intended as a fundamental step to address the Northern leopard frog population trends and to explore the temporal patterns of occupancy, and the underlying population processes of extinction and colonization. To this end we conducted repeat visual surveys each spring and summer from 2009–2013 at 68 wetlands spanning most of the remaining range of Northern leopard frogs in Alberta [13]. We used occupancy modeling to examine seasonal patterns of occupancy and to determine if occupancy was increasing, decreasing or had stabilized over the five-year study. We further examined the underlying extinction and colonization dynamics governing regional patterns of occupancy.

## Materials and Methods

### Study area

**Fig 1:**
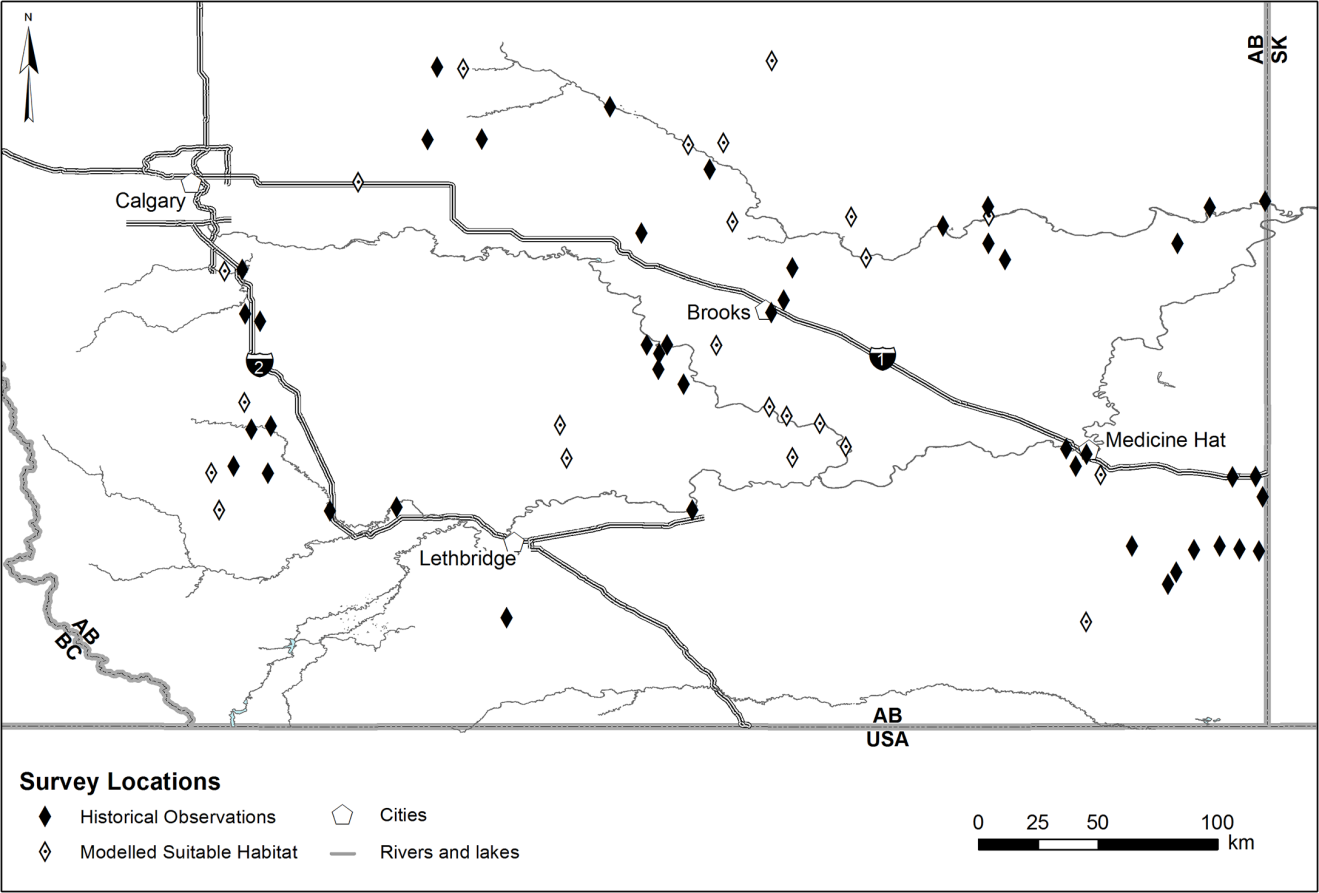
Northern leopard frog visual survey locations in southern Alberta 2009–2013. Black diamonds represent sites where Northern leopard frogs were previously observed during 2005–2008 provincial amphibian surveys (n = 45). White diamonds with center dot represent modelled suitable habitat selected from Habitat Suitability Index models (n = 23). Dark grey lines represent major highways, mid-grey lines are provincial and country borders, and light grey lines represent major drainages in the region. Reprinted from Herpetological Conservation and Biology under a CC BY license, with permission from Des Smith.

Our study area encompassed approximately 56,260 km^2^ of prairie grasslands in southern Alberta, Canada (Fig 1) which represents about 85% of the remaining range of Northern leopard frogs in Alberta [20,21]. Northern leopard frogs occur in patchily distributed populations in this landscape fragmented by roads, agriculture and oil and natural gas development [13,22]. We monitored wetlands because they are places where Northern leopard frogs breed, may forage and overwinter, and where eggs and tadpoles develop.

### Site selection and survey design

We selected 68 sites where Northern leopard frogs had either been observed during 2005–2008 provincial amphibian surveys (n = 45) [20] or where habitat was predicted to be suitable for Northern leopard frogs based upon Habitat Suitability Index (HSI) models (n = 23) developed for Alberta [23,24].

From 2009 to 2013, we used time-constrained repeat visual encounter surveys to locate Northern leopard frogs [24,25]. Each year we conducted four repeat surveys at each site during the spring breeding season (20 April–02 June) and again in the late summer (21 July–30 August) when juveniles are abundant and probability of detection is high [24]. This involved walking and/or wading slowly along the edge of the wetland and among emergent vegetation, visually searching for any life-stage of Northern leopard frog. Spring visual surveys primarily detect breeding adults and non-breeding young of the previous year(s), while most observations during summer surveys are of recently metamorphosed individuals. The timing of our surveys did not include times when tadpoles are abundant as they are difficult to identify and detect using visual survey methods. All sites were located at least 4 km apart to ensure that observations were independent [16,26,27]. Visual surveys at each site were typically an hour in duration but some surveys were shorter if the site was small and could be thoroughly surveyed in less time. For this reason, transects differed in length and survey duration, but each year we surveyed the same section of transect at each site and kept the survey duration constant among years to standardize survey effort at each site. To meet the assumptions of closure we surveyed each site on four successive days [28]. Occasionally it was not possible to conduct all four surveys due to logistic constraints such as weather or road closures.

Detection of anurans is closely linked to environmental conditions such as wind, temperature and moisture [24,29,30]. Failing to account for imperfect detection, i.e. not detecting the survey target when it is present, may cause severe underestimates of occupancy, resulting in inaccurate conclusions regarding occupancy dynamics and studies of habitat association [28]. To address this concern, we measured survey and site-specific covariates such as relative humidity, dew point, air temperature, wind chill, and maximum and average wind speeds at an approximate height of 2 m for 2 min using a Kestrel 3000 Pocket Weather Meter (NK) at the beginning of each survey. Using a waterproof pH/water temperature meter (HI98127 Hanna Instruments) we measured surface water temperature at least twice on each survey at a depth of approximately three inches at 30-minute intervals along the survey transect. To reduce the effects of heterogeneity resulting from observer and time of day effects, we rotated observers and the order sites were surveyed over each consecutive 4-day survey period, and kept survey results confidential (i.e. observers did not know if frogs had been detected by previous observers) [31,32]. We also recorded the survey duration, date and time of day to account for differences in detection probability.

### Statistical approach

We used multi-season occupancy models (PRESENCE 6.1) to explore seasonal patterns of occupancy (i.e., extinction and colonization dynamics between spring and summer within years) and examine the 5-year annual trends separately in spring and summer occupancy of Northern leopard frogs in southern Alberta. Multi-season occupancy models that account for imperfect detection can explicitly model site occupancy (ψ) as well as local colonization (γ) (site transitions from unoccupied to occupied) and extinction (ε) (site transitions from occupied to unoccupied) rates [5,28]. Extinction in this sense can occur if all individuals at a site either die or permanently emigrate, due to between year factors such as decreasing habitat quality, disease, or predation, or within year factors such as seasonal or specific life-stage habitat requirements. Likewise, colonization may occur due to immigration to a new site or shifting seasonal habitat.

To reduce bias of occupancy estimates, we fitted detection probability (*p*) models to determine which covariates most influence detection, and then using the covariate from the top model, we tested specific hypotheses of annual and seasonal occupancy dynamics. We initially analyzed spring and summer seasons together to determine if occupancy estimates were most strongly influenced by seasonal or annual variation and then analyzed spring and summer seasons separately to determine if occupancy was stable, increasing or decreasing in either spring or summer over the 5-year study, and further examined the underlying colonization and extinction dynamics. If occupancy is more strongly affected by season than annual dynamics it would indicate that factors affecting extinction or colonization between seasons (between spring and summer or between summer and spring) such as seasonal habitat selection or seasonal sources of mortality were more important drivers of occupancy than annual variation. We estimated occupancy directly because change in occupancy over time was the primary issue of interest. However, because you can only simultaneously model two of the three parameters (occupancy and extinction or colonization) we choose to directly model occupancy and colonization and used these models to test our hypotheses. We then used the alternate parameterization to directly model occupancy and extinction to obtain extinction estimates, these results are not reported in the tables unless otherwise noted.

Akaike’s information criterion (AIC) was used to select the most parsimonious model, and calculate model weight [33]. We calculated model-averaged parameter estimates from the full-model set and calculated the evidence ratio for balanced model sets. We further discuss only models that have “substantial” support (<2 ΔAIC) [34]. We did not attempt to fit models with higher-order trend lines because there was no reason to suspect non-linear trends based on naïve occupancy estimates and we did not want to risk over-parameterizing our models. There are currently no Goodness-of-Fit tests available for multi-year models; to nonetheless assess if model ranking was sensitive to overdispersion we tested the impact of varying the models’ variance inflation factor (ĉ), using values 1.25, 1.5, 1.75 and 2 [35]. Our model rankings appeared insensitive to overdispersion unless otherwise noted in the results and discussion.

#### Probability of detection models (n = 13)

Spring and summer detection models were fit separately. In each case, we used a general model that allowed rates to vary (both within and among years) with year-specific colonization and extinction: ψ, γ(year), ε(year), p(one of variables described below) and fit standardized covariates thought to influence detection (See reference [36]). We compared 13 models: 10 with each of the following single covariates on probability of detection: air temperature, water temperature, average wind speed, maximum wind speed, wind chill, dew point, humidity, Julian date, survey duration, and time of day. The remaining three models compared constant detection probability against probability of detection that varied with each survey or with year. We calculated the model-averaged probability of detection across years, separately for spring and summer. We only present the top five detection models which jointly comprise the vast majority of the AIC weight (i.e. >99%).

#### General seasonal and annual patterns of occupancy (n = 9)

Models examining changes in occupancy between seasons and among years included the top covariate on probability of detection for each season (as determined by the previous analyses), as well as season as a covariate. To see whether season or year most strongly influenced occupancy, we constructed nine models that allowed occupancy and colonization to vary with season: ψ(season), γ(season) or with year: ψ(year), γ(year) or with year and season: ψ(year & season), γ(year & season) and compared all combinations of these effects (e.g. ψ(season), γ(year & season)).

#### Annual trends in spring and summer occupancy (n = 20)

To examine annual occupancy extinction and colonization dynamics in spring and summer, we compared the following models (n = 10 spring, n = 10 summer) with competing scenarios; increasing or decreasing occupancy trend: ψ(linear), or occupancy varies each year: ψ(year), or occupancy is stable. We modeled possible stable occupancy in four different ways: (1) a true equilibrium model with colonization and extinction specified as constant: ψ(constant), γ(constant) (note: if occupancy and colonization are both constant then extinction must also be constant); (2) a zero turnover model that specifies zero colonization and extinction: (γ = 0, ε = 0); or (3) extinction or colonization decline or increase at a rate that balances each other resulting in no changes in occupancy: ψ(constant), γ(linear), or (4) a trend model that allows occupancy to vary linearly but finds neither an increasing nor decreasing trend: ψ(linear) (i.e. model output for slope of occupancy trend = 0). We also conducted a perturbation analysis to assess the sensitivity of equilibrium occupancy to changes in colonization or extinction rates [3]. We used variance-stabilized sensitivities (VSS) to avoid inaccurate conclusions based on inappropriate scaling (See [3,37] for information).

### Accounting for potential bias

Across species, occupancy tends to be greater in sites with known historic occupancy than in randomly selected sites [31]. Therefore, if the same processes driving changes in occupancy within the entire population (i.e. same rate of extinction and colonization) also occur in sites with biased initial occupancy, an apparent declining trend in occupancy in historic sites may be observed even if regional occupancy is stable [31]. To ensure that any observed trends were not an artifact of site selection criteria, we tested to see if there was evidence of differences in trends in occupancy based upon site selection criteria (i.e. is occupancy declining faster in sites with historic occupancy versus sites picked from the habitat suitability model). We compared models with the competing hypotheses described previously with a model that allowed for differing trends in occupancy depending on site selection type. We did not include site selection criteria as a covariate on colonization or extinction as these estimates should remain unbiased [28].

### Ethics Statement

This research was conducted in compliance with the following permits: Alberta Tourism, Parks and Recreation: RC08SE001, RC09ED005, 11–035, 12–039; EID permits 2009–2012; ASRD permits 39614 and 51679; our protocols were also subject to review by the Calgary Zoo’s Biological Research Review Committee 2008–11. Permission to access private land was obtained from landowners prior to conducting surveys. Private landowners who granted access in this study wish to remain anonymous and specific GPS coordinates cannot be provided as part of that confidentiality.

## Results

We surveyed each of the 68 wetlands an average of 3.88 times each season (spring or summer), each year from 2009–2013. Without accounting for imperfect detection, our naïve estimates of occupancy were 0.35+/-0.02 in the spring and 0.53 +/- 0.01 in the summer.

### Probability of detection

Detection probability was less in spring (0.44 +/- 0.03 SE) than in summer (0.77 +/- 0.02 SE) and decreased when it was cold and windy (Table 1). Wind chill had the strongest influence on both spring (77% AIC weight) and summer (52% AIC weight) detection. Air temperature had the next strongest influence on spring (23% AIC weight) and summer (47% AIC weight) detection. Air temperature and windchill are highly correlated (>98%).

**Table 1:** Top five Northern leopard frog detection probability models for 2009–2013 spring (20 April–02 June) or summer (21 July–30 August) visual surveys in southern Alberta.

Model		-2 LL	K	AIC	ΔAIC	*w*_*i*_
Spring	p(Wchill)	979.02	11	1001.02	0.00	0.77
	p(AT)	981.43	11	1003.43	2.41	0.23
	p(Wmax)	1008.11	11	1030.11	29.09	0.00
	p(Wavg)	1008.43	11	1030.43	29.41	0.00
	p(HM)	1011.68	11	1033.68	32.66	0.00
Summer	p(Wchill)	1042.88	11	1064.88	0.00	0.52
	p(AT)	1043.10	11	1065.10	0.22	0.47
	p(WT)	1052.06	11	1074.06	9.18	0.01
	p(HM)	1052.80	11	1074.80	9.92	0.00
	p(JD)	1053.10	11	1075.10	10.22	0.00

-2 LL = -2 log likelihood; K = number of parameters; AIC = Akaike information criterion; *w*_*i*_ = Akaike weights; *p* = probability of detection; wind chill (Wchill); air temperature (AT); maximum (Wmax) and average (Wavg) wind speed; water surface temperature (WT); humidity (HM); Julian date of survey (JD). Variance inflation factor ĉ = 1.

### General seasonal and annual patterns of occupancy

There were two occupancy models with substantial support (Table 2) and a combined AIC weight of >99%; both of these models included season as a covariate on occupancy. There was roughly equivalent support for models with only season, or season and year as covariates on colonization. There was no support for models that included year, or season and year, as covariates on occupancy, or year as a covariate on colonization. Model-averaged occupancy estimates calculated from the whole model set (Table 3) were less in spring (ψ^Spring^ = 0.40 +/- 0.04) than in summer (ψ^Summer^ = 0.53 +/- 0.05), and year-to-year estimates of colonization were greater than extinction from spring to summer (ɣ^Spring → Summer^ = 0.31 +/- 0.06, 0.27 +/- 0.07, 0.28 +/- 0.06, 0.25 +/- 0.08; 0.25 +/- 0.07 versus ε^Spring → Summer^ = 0.15 +/- 0.05, 0.09 +/- 0.05, 0.11 +/- 0.04, 0.06+/- 0.03, 0.06+/- 0.03), but the opposite was true over the winter (ɣ ^Summer → Spring^ = 0.13 +/- 0.05, 0.10 +/- 0.04, 0.07 +/- 0.03, 0.11 +/- 0.04 versus ε ^Summer → Spring^ = 0.35 +/- 0.05, 0.32 +/- 0.05, 0.29 +/- 0.05, 0.33 +/- 0.05). These results also suggested that over-summer (spring to summer) colonization may have declined over the course of the 5 years of the study so we further explored this relationship by examining trends in spring and summer occupancy dynamics.

**Table 2:** Seasonal and annual Northern leopard frog occupancy dynamics in southern Alberta from 2009–2013 spring (20 April–02 June) and summer (21 July–30 August) visual surveys.

Model	-2 LL	K	AIC	ΔAIC	*w*_*i*_
ψ(season), ɣ(season)	2028.94	7	2042.94	0.0	0.56
ψ(season), ɣ(season&year)	2015.44	14	2043.44	0.5	0.43
ψ(season), ɣ(year)	2039.12	7	2053.12	10.2	0.00
ψ(year), ɣ(season&year)	2009.83	22	2053.83	10.9	0.00
ψ(year), ɣ(season)	2024.46	15	2054.46	11.5	0.00
ψ(year), ɣ(year)	2031.46	15	2061.46	18.5	0.00
ψ(season&year), ɣ(year)	2055.42	7	2069.42	26.5	0.00
ψ(season&year), ɣ(season)	2056.58	7	2070.58	27.6	0.00
ψ(season&year), ɣ(season&year)	2043.20	14	2071.20	28.3	0.00

-2 LL = -2 log likelihood; K = number of parameters; AIC = Akaike Information Criterion; *w*_*i*_ = Akaike weight; ψ = occupancy; ɣ = colonization. Variance inflation factor ĉ = 1.

**Table 3:** Model-averaged parameter estimates from Table 2 models of seasonal and annual Northern leopard frog occupancy in southern Alberta from 2009–2013 visual surveys.

Season		Occ	SE	Transition		Col	SE		Ext	SE
Spr	ψ^2009^	0.40	0.04	Spr^2009^→Sum^2009^	ɣ^1^	0.31	0.06	ε^1^	0.15	0.05
Sum	ψ^2009^	0.53	0.05	Sum^2009^→Spr^2010^	ɣ^2^	0.13	0.05	ε^2^	0.35	0.05
Spr	ψ^2010^	0.40	0.04	Spr^2010^→Sum^2010^	ɣ^3^	0.27	0.07	ε^3^	0.09	0.04
Sum	ψ^2010^	0.53	0.05	Sum^2010^→Spr^2011^	ɣ^4^	0.10	0.04	ε^4^	0.32	0.05
Spr	ψ^2011^	0.40	0.04	Spr^2011^→Sum^2011^	ɣ^5^	0.28	0.06	ε^5^	0.11	0.04
Sum	ψ^2011^	0.53	0.05	Sum^2011^→Spr^2012^	ɣ^6^	0.07	0.03	ε^6^	0.29	0.05
Spr	ψ^2012^	0.40	0.04	Spr^2012^→Sum^2012^	ɣ^7^	0.25	0.08	ε^7^	0.06	0.03
Sum	ψ^2012^	0.53	0.05	Sum^2012^→Spr^2013^	ɣ^8^	0.11	0.04	ε^8^	0.33	0.05
Spr	ψ_2013_	0.40	0.04	Spr^2013^→Sum^2013^	ɣ^9^	0.25	0.07	ε^9^	0.06	0.03
Sum	ψ_2013_	0.53	0.05	-	-	-	-	-	-	-

Model-averaged extinction estimates were obtained using the alternate parameterization of the model. ψ and Occ = occupancy; SE = Standard Error; ɣ and Col = colonization; ε and Ext = extinction; Spr = Spring; Sum = Summer. Variance inflation factor ĉ = 1.

### Annual trends in spring and summer occupancy

**Fig 2:**
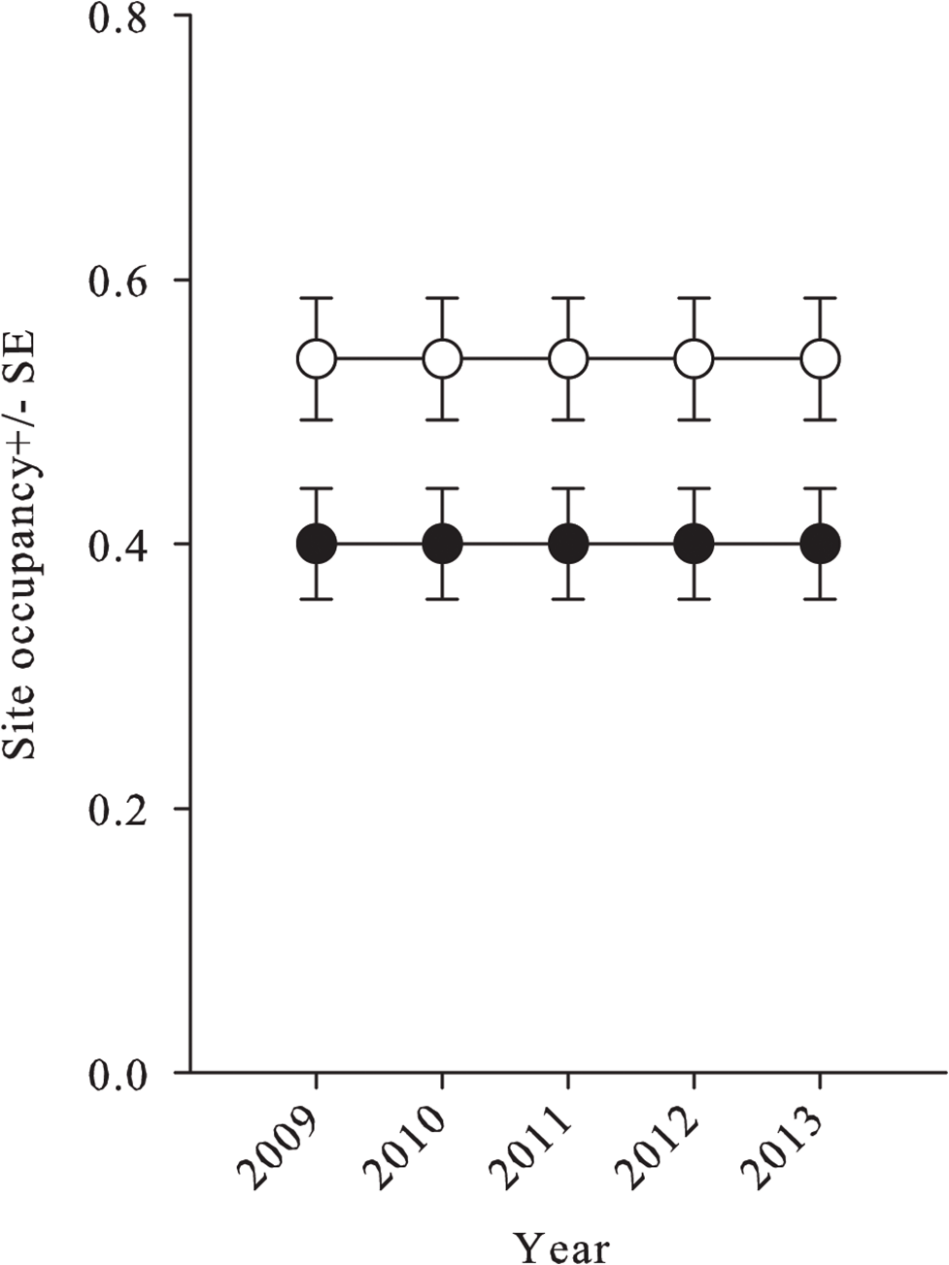
Northern leopard frog occupancy estimates in southern Alberta from 2009–2013 spring and summer visual surveys. Spring (20 April–02 June; black circle) and summer (21 July–30 August; white circle) occupancy was constant with 67% and 66% of the total AIC model weights respectively.

**Fig 3:**
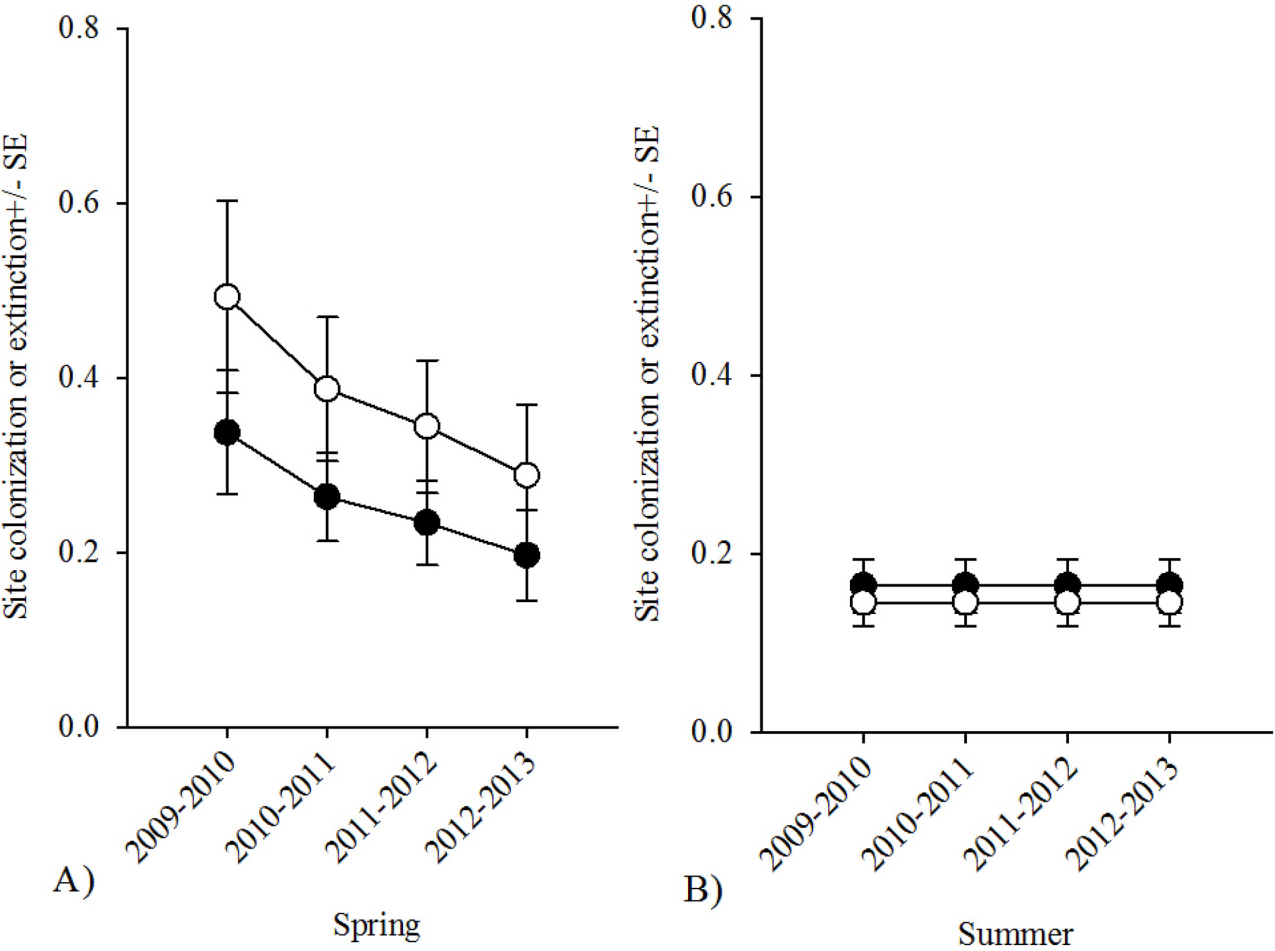
Northern leopard frog extinction and colonization estimates in southern Alberta from 2009–2013 spring and summer visual surveys. A) Spring (20 April–02 June) colonization (black circle) and extinction (white circle) both declined with 53% of the AIC model weights and B) Summer (21 July–30 August) colonization (black circle) and extinction (white circle) were constant with 48% of the model weights.

There were three occupancy trend models for spring with substantial support (Table 4). Two of these models were constant occupancy models (37 and 18% model weights respectively; Fig 2) and the third model demonstrated a slight increasing linear trend in spring occupancy (14% model weight). Overall there was 2.5 times more support for models with constant occupancy than increasing linear occupancy and virtually no support for models with occupancy that varied with year based on the evidence ratio. There was more support for models with declining spring colonization and extinction than constant or yearly varying colonization and extinction (2.0 and 2.8 times more likely respectively; Table 4, Fig 3A). However, at ĉ = 2 there was roughly equal support for the two constant occupancy models (based on the ∆AIC) and roughly equal model weight for the constant and declining spring colonization models (32% and 31% model weight respectively). There was no support (<1% model weight) for models with unchanging site occupancy (i.e. no extinction and colonization).

**Table 4:** Annual Northern leopard frog occupancy trends from 2009–2013 in spring (20 April–02 June) and summer (21 July–30 August) in southern Alberta.

	Model	-2 LL	K	AIC	ΔAIC	*w*_*i*_
Spring	ψ(constant), ɣ(linear)	985.98	5	995.98	0.00	0.37
	ψ(constant), ɣ(constant)	989.37	4	997.37	1.39	0.18
	ψ(linear), ɣ(linear)	985.91	6	997.91	1.93	0.14
	ψ(constant), ɣ(year)	984.28	7	998.28	2.3	0.12
	ψ(linear), ɣ(constant)	988.72	5	998.72	2.74	0.09
	ψ(linear), ɣ(year)	984.25	8	1000.25	4.27	0.04
	ψ(year), ɣ(year)	979.02	11	1001.02	5.04	0.03
	ψ(year), ɣ(linear)	983.42	9	1001.42	5.44	0.02
	ψ(year), ɣ(constant)	988.66	8	1004.66	8.68	0.00
	ψ, ɣ(zero), ε(zero)	1078.76	5	1088.76	92.78	0.00
Summer	ψ(constant), ɣ(constant)	1048.44	4	1056.44	0.00	0.32
	ψ(constant), ɣ(linear)	1047.28	5	1057.28	0.84	0.21
	ψ(constant), ɣ(year)	1044.16	7	1058.16	1.72	0.13
	ψ(linear), ɣ(constant)	1048.34	5	1058.34	1.90	0.12
	ψ(linear), ɣ(linear)	1046.91	6	1058.91	2.47	0.09
	ψ(linear), ɣ(year)	1043.94	8	1059.94	3.50	0.06
	ψ(year), ɣ(constant)	1044.59	8	1060.59	4.15	0.04
	ψ(year), ɣ(linear)	1043.78	9	1061.78	5.34	0.02
	ψ(year), ɣ(year)	1042.88	11	1064.88	8.44	0.00
	ψ, ɣ(zero), ε(zero)	1389.78	5	1399.78	343.34	0.00

-2 LL = -2 log likelihood; K = number of parameters; AIC = Akaike Information Criterion; *w*_*i*_ = Akaike weight; ψ = occupancy; ɣ = colonization; ε = extinction. Variance inflation factor ĉ = 1.

There were four summer occupancy models with substantial support (Table 4), three of which were constant occupancy models with a combined AIC weight of 66% (Fig 2) and the fourth suggested that summer occupancy may have declined over the course of the study (12% AIC weight). There was 2.4 times more support (evidence ratio) for models with constant occupancy opposed to declining occupancy and there was slightly more support for models with constant colonization (Fig 3B) vs. models with declining extinction and colonization rates or yearly varying colonization and extinction (1.5 and 2.5 times more likely respectively). Similar to spring, there was virtually no support for models with unchanging site occupancy (<1%). We calculated the model-weighted parameter estimates for the whole model set for spring and summer. Occupancy was 0.41 +/- 0.05, colonization was 0.21 +/- 0.5, and extinction was 0.30 +/- 0.07 in spring, and occupancy was 0.53 +/- 0.05, colonization was 0.16 +/- 0.4, and extinction was 0.14 +/- 0.03 for summer. The variance-stabilized sensitivity metrics from our perturbation analysis based upon estimates of γ and ε from spring and summer equilibrium models were: Spring VSSγ = 0.98, VSSε = -0.77, and Summer VSSγ = 1.10, VSSε = -1.23. Thus spring |VSSγ|>|VSSε| but summer |VSSγ|<|VSSε|.

### Accounting for potential bias

Although occupancy was greater in sites selected based upon historic observations (spring 0.47 +/- 0.05 SE; summer 0.67 +/- 0.05 SE) than in sites selected using modelled suitable habitat (spring 0.26 +/- 0.06 SE; summer 0.29 +/- 0.07) there was not substantial support for the model that tested whether occupancy trends differed between historic and model-selected sites (Table 5; evidence ratio of constant over different trends: 3.0 spring, 65.0 summer); thus site selection criteria were not seen as a significant confound to further analyses.

**Table 5:** Annual Northern leopard frog occupancy trends with site selection criteria (SSC) from 2009–2013 in spring (20 April–02 June) and summer (21 July–30 August) in southern Alberta.

	Model	-2 LL	K	AIC	ΔAIC	*w*_*i*_
Spring	ψ(SSC constant), ɣ(year)	976.88	8	992.88	0.00	0.49
	ψ(SSC linear), ɣ(year)	976.78	9	994.78	1.90	0.19
	ψ(SSC year), ɣ(year)	971.08	12	995.08	2.20	0.16
	ψ(SSC different linear), ɣ(year)	977.08	9	995.08	2.20	0.16
	ψSSC, ɣ(none), ε(none)	1072.70	6	1084.7	91.82	0.00
Summer	ψ(SSC constant), ɣ(year)	1029.75	8	1045.75	0.00	0.65
	ψ(SSC linear), ɣ(year)	1029.23	9	1047.23	1.48	0.31
	ψ(SSC year), ɣ(year)	1028.09	12	1052.09	6.34	0.03
	ψ(SSC diff linear), ɣ(year)	1035.96	9	1053.96	8.21	0.01
	ψSSC, ɣ(zero), ε(zero)	1384.80	6	1396.80	351.05	0.00

-2 LL = -2 log likelihood; K = number of parameters; AIC = Akaike Information Criterion; *w*_*i*_ = Akaike weight; ψ = occupancy; ɣ = colonization; ε = extinction. Variance inflation factor ĉ = 1.

## Discussion

Although our understanding is improving, information on regional occupancy trends and the underlying colonization and extinction dynamics is lacking for many amphibian species. This knowledge deficit may hinder our ability to detect changes in occupancy of at-risk species and thus intervene if conservation action is required or measure the effectiveness of management decisions. In Canada, one of the criteria for listing species under COSEWIC guidelines, and therefore prompting conservation actions, is a continued decline in extent of occurrence or area of occupancy [38]. Understanding seasonal changes in occupancy can help target research questions to help identify causes of declining occupancy or sources of mortality that may hinder recovery.

Our analyses revealed that regional occupancy increased from spring to summer and declined over the winter. This seasonal pattern of occupancy was likely due to several factors such as differing seasonal habitat requirements, annual recruitment, overwinter mortality and dispersal. In general, occupancy was low in the spring, perhaps due to a shortage of suitable breeding ponds causing adult frogs to congregate at a limited number of sites, or because local overwinter extinction could result in the death of all breeding age adults within a reasonable proximity to a suitable breeding pond. Colonization rate increased between spring and summer perhaps because increased population density due to recruitment triggers dispersal, resulting in the occupation of more sites in the summer [27]. It is also possible that Northern leopard frogs may occupy a greater diversity of habitats in the summer (i.e. young-of-year may remain near natal ponds until fall migration [16] while post-breeding adults may disperse to nearby foraging or overwintering grounds) [27]. Extinction was greater than colonization over the winter and occupancy was less in the spring than in the summer. This pattern of extinction and colonization may be caused by high overwinter mortality or because some of the sites occupied by individuals dispersing in the summer may be sink habitats that are not capable of maintaining Northern leopard frogs throughout the year [13]. Occupancy may have been greater in summer because there was more available summer foraging habitat or because higher abundance caused density-dependant dispersal to less favorable habitat which may or may not act as a dispersal sink [39]. These hypotheses should be tested once appropriate data has been collected.

Although Northern leopard frog occupancy varied with season, occupancy was apparently stable (constant) within each season over the five year study (i.e. within-year occupancy varied but there was little variation in occupancy in a given season among years). Stable occupancy can occur by way of three different underlying extinction and colonization patterns: (1) a true equilibrium with constant colonization and extinction, (2) zero turnover in occupancy status of a site (no colonization or extinction), or (3) extinction or colonization decline or increase at a rate that balances resulting in no changes in occupancy [28]. Our results suggested that spring occupancy was constant due to a declining rate of extinction balanced by declining colonization and summer occupancy was at equilibrium, with constant rates of extinction and colonization. However, the model ranking of the spring model with declining colonization and extinction was sensitive to the effects of overdispersion thus we cannot discount that constant spring occupancy may also have been at equilibrium. Our perturbation analysis revealed that in spring, occupancy is more sensitive to changes in colonization than extinction, whereas in summer, occupancy is more sensitive to changes in extinction than colonization. Therefore, management decisions and conservation efforts that are aimed at increasing colonization in the spring or decreasing extinction in the summer are more likely to increase occupancy [3]. Thus, approaches aimed at decreasing overwinter mortality (e.g. by removing predatory fish or increasing dissolved oxygen) or increasing spring colonization (e.g. through improvement of, or improved access to, breeding habitat or translocation of individuals to new sites) may be most effective strategy for increasing occupancy. However, because summer occupancy is already quite high and extinction rates low, it may be difficult to influence summer annual extinction rate. These sensitivity metrics assume that the system has reached equilibrium, but even if that were not the case in our system in the spring, valuable insight may be gained regarding the influence of rate parameters for systems not currently at equilibrium [3].

The mechanisms driving colonization and extinction in amphibians are likely complex. If colonization and extinction were concurrently declining in spring there are few simple explanations. In general, factors that tend to increase site colonization usually decrease the probability of extinction; for example, increased connectivity typically leads to increased dispersal and colonization of new sites as well as decreased probability of extinction due to immigration from nearby occupied sites (the rescue effect) [40]. However, there are scenarios where factors that tend to decrease site colonization may also decrease extinction. For example, decreased connectivity could also result in decreased probability of extinction if it results in lower occupancy of sink habitats or disease transmission [39]. Similarly, decreased reproductive success and abundance, perhaps caused by habitat degradation or declining water levels, could increase or decrease extinction. Recently metamorphosed and sub-adult Northern leopard frogs are the primary agents of dispersal and colonizers of new sites [16], therefore declining reproductive success could lead to decreased dispersal and lower colonization rates [41]. Declining reproductive rates could lead to lower abundance and thus one would predict higher extinction rates [42], however, lower abundance could lead to lower probability of extinction if it reduces density-dependant effects such as disease transmission [43].

This is the first rigorous assessment of occupancy dynamics for Northern leopard frogs not only for Alberta, but for the species in general. Previous surveys for Northern leopard frogs in Alberta only provided a snapshot of species distribution without providing information of the population dynamics or the underlying processes driving them and did not account for imperfect detection. Although our study revealed that occupancy was apparently stable over the 5 year study period, due to the large scope of our study and the prohibitive cost and effort, we could not also measure abundance. It is possible that abundance was increasing without increased colonization of sites due to decreased habitat connectivity or lack of suitable habitat or that abundance was declining but had not reached an extinction threshold. We should also note that short-term trends may not be representative of longer term dynamics [44], and changes in occupancy may be happening at a rate too slow to be detected within our study’s five-year time-frame [45].

Amphibians are thought to be particularly susceptible to the effects of habitat degradation and fragmentation associated with development due to their typically limited dispersal capabilities and specific habitat requirements [41,46]. Northern leopard frogs are also predicted to be especially vulnerable to the effects of climate change in recent projections for Alberta [47]. It is estimated that Alberta has already lost over two thirds of wetlands in settled areas of the province [48] and prairie wetlands are slow to recover from development activities [49]. Although Northern leopard frog juveniles will occasionally disperse long distances, habitat loss and fragmentation make the natural re-colonization of extinct sites increasing less likely. The goal of the 2010–2015 Alberta Northern leopard frog recovery plan is to “achieve well-distributed and self-sustaining populations of Northern leopard frogs throughout their historic range in Alberta” [13]. Although conservation efforts, such as translocation and habitat protection are on-going, further population augmentation and translocation combined with protection of key breeding, foraging and over-wintering habitat may be required in order to reach this conservation goal.

This paper focuses on what we see as a first, fundamental step: establishing what temporal patterns exist in the occupancy, extinction and colonization dynamics of Northern Leopard frogs in Alberta, especially on a landscape level. The next step should be to gain an understanding about what factors are influencing occupancy dynamics. There are many different aspects of the landscape (e.g. area/edge, shape, aggregation, watershed connectivity, land cover, land use, distance to roads or water bodies etc.) and site-specific habitat characteristics (e.g. water body type, water quality, presence of fish or disease etc.) which might influence occupancy trends, and true drivers are most likely to be identified only if a comprehensive set of factors is considered. Future research should be focused on the possible causes of low spring occupancy, such as high over-winter mortality or a lack of suitable breeding ponds; this may help us understand factors limiting species recovery in this region.

Classic metapopulation theory is often applied to help understand the population dynamics of many species but it is not appropriate for all species of amphibians [50–52]. The consequences of this are that we may underestimate the scale of the metapopulation effect and overemphasize the importance of regional rather than local population dynamics to long-term persistence [52]. Although some conservation planning does occur at the provincial scale (e.g. Alberta Northern Leopard Frog Recovery Team), most conservation action is focused on individual sites rather than the scale of the landscape. One way that metapopulation theory is applied to amphibian conservation relates to the “ponds as patches” concept that suggests that by protecting breeding ponds we are protecting the habitat critical to the species survival; however, our study provides evidence that events occurring outside of the breeding pond, such as migration and over-wintering survival, may play an important role in population dynamics.

These results have important implications for conservation strategies for Northern leopard frogs and provide insights into amphibian population dynamics that may be of broader significance for other species. Many studies of population dynamics only consider occupancy in a single year or season, and thus do not reveal the underlying complexity of seasonal and annual occupancy. Our study design and statistical approach could be used to assess the occupancy dynamics of any species that experiences differences in annual and seasonal occupancy, helping to develop informed conservation strategies that target the seasonal vital rates most likely to influence occupancy.
